# Interleukin-6 and Asymmetric Dimethylarginine Are Associated with Platelet Activation after Percutaneous Angioplasty with Stent Implantation

**DOI:** 10.1371/journal.pone.0122586

**Published:** 2015-03-25

**Authors:** Thomas Gremmel, Thomas Perkmann, Christoph W. Kopp, Daniela Seidinger, Beate Eichelberger, Renate Koppensteiner, Sabine Steiner, Simon Panzer

**Affiliations:** 1 Division of Angiology, Department of Internal Medicine II, Medical University of Vienna, Vienna, Austria; 2 Department of Laboratory Medicine, Medical University of Vienna, Vienna, Austria; 3 Department of Blood Group Serology and Transfusion Medicine, Medical University of Vienna, Vienna, Austria; 4 Center for Vascular Medicine—Angiology, Cardiology and Vascular Surgery, Park Hospital Leipzig, Leipzig, Germany; University Hospital Medical Centre, GERMANY

## Abstract

Data linking *in vivo* platelet activation with inflammation and cardiovascular risk factors are scarce. Moreover, the interrelation between endothelial dysfunction as early marker of atherosclerosis and platelet activation has not been studied, so far. We therefore sought to investigate the associations of inflammation, endothelial dysfunction and cardiovascular risk factors with platelet activation and monocyte-platelet aggregate (MPA) formation in 330 patients undergoing angioplasty with stent implantation for atherosclerotic cardiovascular disease. P-selectin expression, activation of glycoprotein IIb/IIIa and MPA formation were determined by flow cytometry. Interleukin (IL)-6, high sensitivity C-reactive protein and asymmetric dimethylarginine (ADMA) were measured by commercially available assays. IL-6 was the only parameter which was independently associated with platelet P-selectin expression and activated GPIIb/IIIa as well as with leukocyte-platelet interaction in multivariate regression analysis (all p<0.05). ADMA was independently associated with GPIIb/IIIa activation (p<0.05). Patients with high IL-6 exhibited a significantly higher expression of P-selectin than patients with low IL-6 (p=0.001), whereas patients with high ADMA levels showed a more pronounced activation of GPIIb/IIIa than patients with low ADMA (p=0.003). In conclusion, IL-6 and ADMA are associated with platelet activation after percutaneous angioplasty with stent implantation. It remains to be established whether they act prothrombotic and atherogenic themselves or are just surrogate markers for atherosclerosis with concomitant platelet activation.

## Introduction

Detrimental platelet activation plays a pivotal role in the development of acute ischemic events [1]. Following atherosclerotic plaque rupture, platelets adhere to exposed subendothelial structures of the injured vessel wall, and initiate clot formation thereby leading to further platelet recruitment and activation with subsequent vessel occlusion. However, it has been shown that even patients with stable atherosclerosis exhibit higher levels of platelet activation than healthy individuals [2], and that the extent of platelet activation in these patients is a strong predictor of future ischemic events [3]. Since atherosclerosis is increasingly recognized as a chronic inflammatory disease, markers of inflammation as well as factors promoting plaque formation may be linked to the extent of platelet activation [4]. Indeed, previous studies reported an association of inflammation and cardiovascular risk factors with on-treatment platelet reactivity. It has been shown that patients with high levels of Interleukin (IL)-6 and C-reactive protein (CRP) exhibit a worse response to antiplatelet therapy with aspirin and clopidogrel [5–8]. Other studies revealed an inadequate response to antiplatelet therapy in patients with advanced age [9], obesity [10, 11], diabetes [12, 13] and chronic kidney disease [14, 15]. However, most of these studies focused on agonists’-inducible platelet reactivity. Consequently, data linking *in vivo* platelet activation with inflammation and cardiovascular risk factors are scarce. Moreover, the interrelation between endothelial dysfunction as early marker of atherosclerosis and platelet activation has not been studied, so far. We therefore sought to investigate the associations of inflammation, endothelial dysfunction and cardiovascular risk factors with platelet activation and monocyte-platelet aggregate (MPA) formation in patients undergoing angioplasty with stent implantation for cardiovascular disease.

## Materials and Methods

### Study Population

The study population comprised 330 patients undergoing angioplasty and stenting for atherosclerotic cardiovascular disease. Clinical and laboratory characteristics of the overall study population are given in Table 1.

**Table 1:** Clinical and laboratory patient characteristics.

Characteristics	n = 330
**Demographics**	
Age, years	65 (57–74)
Male sex	216 (65.5)
BMI, kg/m^2^	27 (24.2–29.7)
**Medical history**	
Hypertension	296 (89.7)
Hyperlipidemia	308 (93.3)
Diabetes mellitus	106 (32.1)
Active smoking	140 (42.4)
**Laboratory data**	
Hemoglobin, g/dl	13.1 (12–14.3)
White blood cell count, G/l	8.4 (7–10.4)
Platelet count, G/l	209 (176–251)
Serum creatinine, mg/dl	1 (0.9–1.2)
High sensitivity CRP, mg/dl	0.81 (0.33–1.82)
Interleukin-6, pg/mL	15.74 (9.81–27.7)
Asymmetric dimethylarginine, μmol/L	0.86 (0.6–1.34)
**Medication pre-intervention**	
Clopidogrel	316 (95.8)
Prasugrel	14 (4.2)
Aspirin	330 (100)
Statins	315 (95.5)
ACE inhibitors/ARB	285 (86.4)
Beta blockers	231 (70)
Proton pump inhibitors	178 (53.9)
Calcium channel blockers	96 (29.1)

Continuous data are shown as median (interquartile range). Dichotomous data are shown as n (%).

BMI, body mass index; CRP, C-reactive protein; ACE inhibitors, angiotensin converting enzyme inhibitors; ARB, angiotensin receptor blockers.

The median age was 65 years (interquartile range 57–74 years) and 65.5% of the study population were male. Hypertension, hypercholesterolemia, diabetes and smoking were seen in 89.7%, 93.3%, 32.1% and 42.4% of the patients, respectively. All patients received daily aspirin (100mg/d) and thienopyridine therapy (75 mg clopidogrel/d or 10 mg prasugrel/d). One hundred seventy-one (51.8%), 121 (36.7%), and 38 patients (11.5%) had peripheral, coronary and cerebrovascular interventions, respectively. Among the patients treated with peripheral angioplasty, iliac, femoropopliteal and crural arteries were intervened in 39 (22.8%), 139 (81.3%), and 7 patients (4.1%), respectively. Among the patients treated with coronary angioplasty, left anterior descending artery, left circumflex artery and right coronary artery were intervened in 58 (47.9%), 34 (28.1%), and 50 patients (41.3%), respectively. All patients with cerebrovascular intervention underwent angioplasty and stenting of the internal carotid artery.

Exclusion criteria were a known aspirin or thienopyridine intolerance (allergic reactions, gastrointestinal bleeding), a therapy with vitamin K antagonists (warfarin, phenprocoumon, acenocoumarol), treatment with ticlopidine, dipyridamol or nonsteroidal antiinflammatory drugs, a family or personal history of bleeding disorders, malignant paraproteinemias, myeloproliferative disorders or heparin-induced thrombocytopenia, severe hepatic failure, known qualitative defects in thrombocyte function, a major surgical procedure within one week before enrollment, a platelet count <100.000 or >450.000/μl and a hematocrit <30%.

The study protocol was approved by the Ethics Committee of the Medical University of Vienna in accordance with the Declaration of Helsinki and written informed consent was obtained from all study participants.

### Blood sampling

Blood was drawn one day after the percutaneous intervention into 3.8% sodium citrate Vacuette tubes (Greiner Bio-One; 9 parts of whole blood, 1 part of sodium citrate 0.129 M/L) for whole blood flow cytometry and determination of asymmetric dimethylarginine (ADMA), and into serum tubes (Greiner Bio-One) for measurements of interleukin (IL)-6 and high sensitivity CRP (hsCRP), as previously described [16].

### Measurement of interleukin (IL)-6 and high sensitivity C-reactive protein (hsCRP)

The IL-6 antigen levels were measured using the Elecsys IL-6 kit (Roche Diagnostics) on the ECL technology based COBAS e411 (Roche Diagnostics). The lower detection limit of this system is 1.5 pg/mL. The reported intra- and inter-assay coefficients of variation are typically lower than 6%.

The hsCRP level was measured using fully automated particle enhanced immuno-nephelometry (N high-sensitivity CRP, Dade Behring, Marburg, Germany) on a Behring nephelometer II (BN Systems, Orchard Park, NY).

### Measurement of asymmetric dimethylarginine (ADMA)

ADMA levels were determined with a commercially available enzyme-linked immunosorbent assay (DLD Diagnostika, Hamburg, Germany) according to the manufacturer’s instructions.

### Determination of P-selectin expression and glycoprotein (GP) IIb/IIIa activation

The expression of P-selectin and the binding of the monoclonal antibody (mAb) PAC-1 to activated GPIIb/IIIa were determined in citrate-anticoagulated blood, as previously published [3]. In brief, whole blood was diluted in phosphate-buffered saline (PBS) to obtain 20 x 10^3^ platelets and incubated for 10 min. The platelet population was identified by staining with anti-CD42b (clone HIP1, allophycocyanin labeled; Becton Dickinson (BD), San Jose, CA, USA), and expression of activated GPIIb/IIIa and P-selectin were determined by the binding of the mAbs PAC-1-fluorescein (BD) and anti-CD62p-phycoerythrin (PE; clone CLBThromb6; Immunotech, Beckman Coulter, Fullerton, CA, USA), respectively. After 15 min of incubation in the dark, the reaction was stopped by adding 500 mL PBS and samples were acquired immediately on a FACS Calibur flow cytometer (BD) with excitation by an argon laser at 488 nm and a red diode laser at 635 nm at a rate of 200–600 events per second. Platelets were gated in a side scatter versus FL3 dot plot. A total of 10000 events were acquired within this gate. The gated events were further analyzed in histograms for FL-1 and FL-2 for PAC-1 and P-selectin, respectively. Standard BD calibrite beads were used for daily calibration of the cytometer.

### Determination of monocyte-platelet aggregate (MPA) formation

MPA formation was determined as previously described [17]. In brief, 100 μL of citrate-anticoagulated whole blood was stained with saturating concentrations of the following fluorochrome-conjugated mAbs: allophycocyanin (APC)-labeled mAb for the constitutive platelet marker CD42b (glycoprotein Ib of von Willebrand factor receptor complex), PECy5-labeled mAb for monocyte CD14 (endotoxin receptor) and corresponding isotype controls (all from BD). After 10 min of pre-incubation with antibodies in the dark at room temperature, samples were fixed and erythrolyzed with Optilyse B (Instrumentation Laboratories). Flow cytometry was performed on a FACSCalibur (BD) flow cytometer. Acquisition was stopped when 3000 CD14+ events were acquired. Monocytes were identified by gating CD14+ events, and all additional analyses were performed on this population. The negative and positive delineator were determined by gating 2% background staining on the isotype control fluorescence. The percentage of MPAs characterized by the relative number of monocytes co-expressing the constitutive platelet marker CD42b (CD14+/CD42b+) was determined.

### Statistical analysis

Statistical analysis was performed using the Statistical Package for Social Sciences (IBM SPSS version 21, Armonk, New York, USA). The Kolmogorov-Smirnov test was used to test for normal distribution. Variables with skewed distribution were log-transformed for regression analyses. After log transformation skewed variables were normally distributed. Median and interquartile range of continuous variables are shown. Categorical variables are given as number (%). We performed Mann Whitney U tests to detect differences of continuous variables. Univariate and multivariate linear regression analyses were used to assess the associations of inflammatory markers, ADMA and cardiovascular risk factors with platelet activation and MPA formation. Two-sided P-values <0.05 were considered statistically significant.

## Results

In univariate analyses, IL-6 and hs-CRP were significantly associated with P-selectin expression; IL-6, ADMA, age, platelet count, white blood cell count (WBC), and serum creatinine were significantly associated with activated GPIIb/IIIa; IL-6, platelet count and female sex were significantly associated with MPA formation (all p<0.05).

The associations of age, sex, body mass index, hypertension, hyperlipidemia, diabetes, active smoking, platelet count, WBC, IL-6, hsCRP, serum creatinine and ADMA with P-selectin expression, GPIIb/IIIa activation and MPA formation were estimated in a multivariate linear regression model. Thereby, IL-6 was the only parameter which was independently associated with both parameters of platelet activation (P-selectin expression and activated GPIIb/IIIa) as well as with leukocyte-platelet interaction (all p<0.05; Table 2). ADMA was independently associated with activation of the fibrinogen receptor GPIIb/IIIa (p = 0.02), whereas the platelet count (p<0.001) and active smoking (p = 0.04) were independently linked to MPA formation (Table 2).

**Table 2:** Regression coefficients (B), confidence intervals (CI), and p-values (p) of multivariate regression analyses of age, sex, body mass index (BMI), hypertension, hyperlipidemia, diabetes, active smoking, platelet count, white blood cell count (WBC), log transformed interleukin-6 (log IL-6),log transformed high-sensitivity C-reactive protein (log hsCRP), log transformed serum creatinine (log creatinine), and log transformed asymmetric dimethylarginine (log ADMA) for surface expression of P-selectin, activation of glycoprotein (GP) IIb/IIIa, and formation of monocyte-platelet aggregates (MPA).

	P-selectin (MFI)			Activated GPIIb/IIIa (MFI)			MPA formation (%)		
	B	CI	P	B	CI	p	B	CI	P
**Age**	0.01	-0.01–0.03	0.3	0.01	-0.01–0.02	0.3	-0.1	-0.2–0.01	0.06
**Female sex**	0.03	-0.4–0.5	0.9	0.09	-0.2–0.4	0.5	0.7	-1.6–3	0.5
**BMI**	-0.02	-0.07–0.03	0.5	-0.01	-0.03–0.02	0.7	-0.01	-0.3–0.2	0.9
**Hypertension**	-0.3	-1–0.4	0.4	0.1	-0.3–0.5	0.5	-2.4	-5.9–1.2	0.2
**Hyperlipidemia**	0.2	-0.6–1	0.6	0.04	-0.4–0.5	0.9	2.6	-1.3–6.5	0.2
**Diabetes**	0.3	-0.1–0.7	0.1	0.2	-0.08–0.4	0.2	-0.4	-2.5–1.7	0.7
**Smoking**	0.3	-0.1–0.8	0.2	-0.05	-0.3–0.2	0.7	-2.3	-4.6 –-0.1	0.04
**Platelet count**	0	-0.004–0.003	0.8	-0.001	-0.003–0.001	0.4	0.05	0.03–0.07	<0.001
**WBC**	-0.06	-0.1–0.01	0.1	-0.03	-0.08–0.01	0.1	-0.3	-0.7–0.1	0.2
**Log IL-6**	1.1	0.4–1.7	0.003	0.4	0.05–0.8	0.03	3.6	0.1–7	0.04
**Log hsCRP**	0.2	-0.2–0.7	0.3	0.05	-0.2–0.3	0.7	0.4	-1.7–2.6	0.7
**Log creatinine**	-0.05	-2.1–2	0.9	0.4	-0.8–1.6	0.5	-1.6	-11.5–8.4	0.8
**Log ADMA**	0.04	-0.5–0.6	0.9	0.4	0.08–0.7	0.02	0.9	-1.8–3.5	0.5

MFI, mean fluorescence intensity.

Levels of ADMA were significantly lower in prasugrel-treated patients than in patients receiving clopidogrel (median [interquartile range]: 0.66 μmol/L [0.32–0.79 μmol/L] vs. 0.91 μmol/L [0.61–1.4 μmol/L]; p = 0.003). Levels of IL-6, P-selectin expression, activated GPIIb/IIIa and MPA formation did not differ significantly between clopidogrel- and prasugrel treated patients (all p>0.1). An additional multivariate regression analysis including only clopidogrel-treated patients did not change the results.

All patients treated with peripheral angioplasty had intermittent claudication. Among the patients treated with coronary angioplasty (n = 121; 36.7%), 44 (36.4%), 41 (33.9%) and 36 (29.7%) had stable angina, unstable angina/non ST-segment elevation myocardial infarction (UA/NSTEMI) and ST-segment elevation myocardial infarction (STEMI), respectively. As expected, patients with an acute coronary syndrome (ACS; UA/NSTEMI or STEMI) had significantly higher levels of hsCRP than patients without ACS (median [interquartile range]: 1.56 mg/dl [0.45–4.23 mg/dl] vs. 0.74 mg/dl [0.31–1.52 mg/dl]; p<0.001). Levels of IL-6 and ADMA, platelet activation parameters (P-selectin, activated GPIIb/IIIa) and MPA formation did not differ significantly between patients without and with ACS (all p>0.05). The adjustment for ACS in the multivariate linear regression model did not change the results.

**Fig 1:**
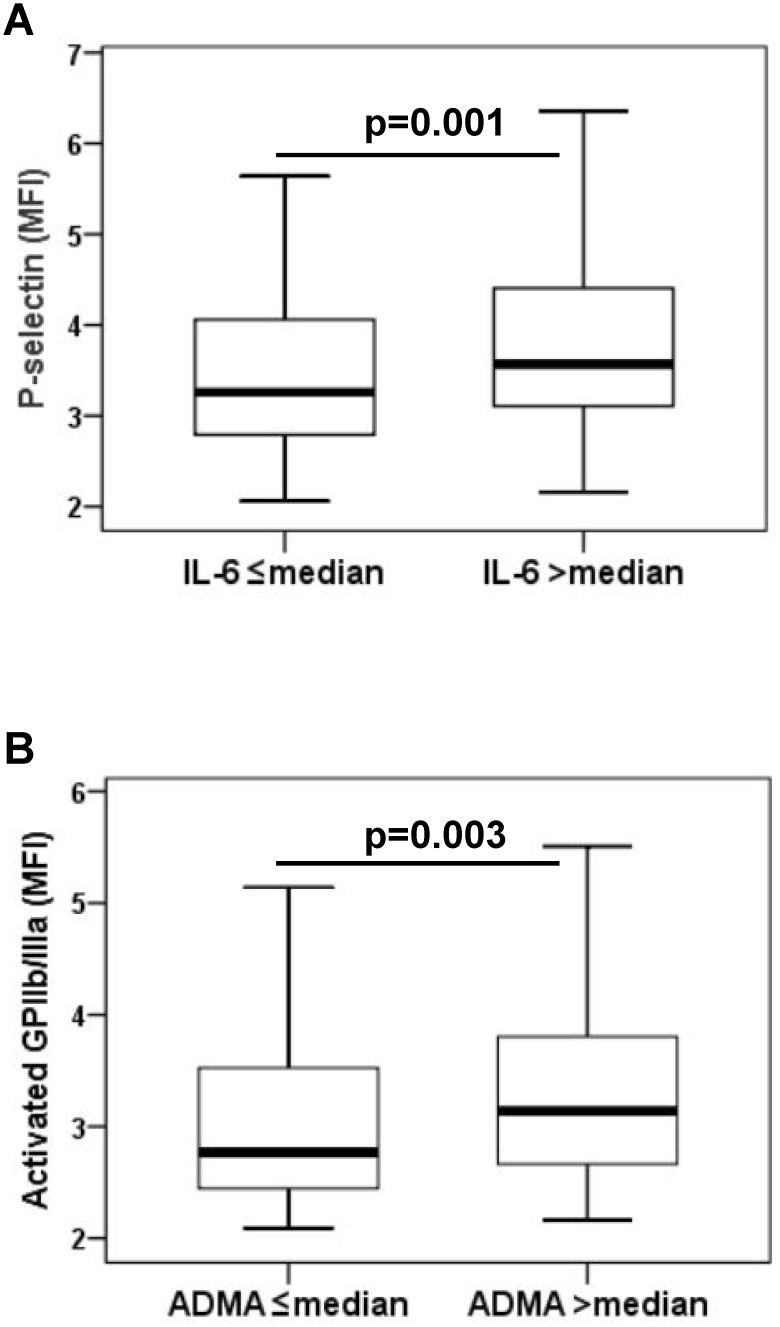
Platelet surface expression of P-selectin and activated glycoprotein (GP) IIb/IIIa. **(A)** Platelet surface expression of P-selectin in patients with high (>median) vs. low (≤median) interleukin (IL)-6 levels. **(B)** Platelet surface expression of activated GPIIb/IIIa in patients with high (>median) vs. low (≤median) asymmetric dimethylarginine (ADMA) levels. The boundaries of the box show the lower and upper quartile of data, the line inside the box represents the median. Whiskers are drawn from the edge of the box to the highest and lowest values that are outside the box but within 1.5 times the box length. MFI, mean fluorescence intensity.

In a second step, IL-6 levels >median (>15.74 pg/mL) were defined as high IL-6 and IL-6 levels ≤median (≤15.74 pg/mL) were defined as low IL-6. The platelet count did not differ significantly between patients with high and low IL-6 (p = 0.8). Patients with high IL-6 exhibited a significantly higher platelet surface expression of P-selectin than patients with low IL-6 (3.4 MFI [2.9–4.2 MFI] vs. 3.1 MFI [2.6–3.9 MFI], p = 0.001; Fig. 1A).

Similarly, ADMA levels >median (>0.86 μmol/L) were defined as high ADMA and ADMA levels ≤median (≤0.86 μmol/L) were defined as low ADMA. Patients with high ADMA showed a more pronounced activation of GPIIb/IIIa than patients with low ADMA (2.9 MFI [2.5–3.6] vs. 2.6 MFI [2.3–3.3], p = 0.003; Fig. 1B).

Age, female sex, active smoking, WBC, and serum creatinine were independently associated with high IL-6, while none of the tested patient characterictics was independently associated with high ADMA (Table 3).

**Table 3:** Regression coefficients (B), confidence intervals (CI), and p-values (p) of multivariate regression analysis of age, sex, body mass index (BMI), hypertension, hyperlipidemia, diabetes, active smoking, platelet count, white blood cell count (WBC), and log transformed serum creatinine (log creatinine) for high interleukin-6 levels (high IL-6) and high asymmetric dimethylarginine (high ADMA).

	High IL-6			High ADMA		
	B	CI	P	B	CI	p
**Age**	0.01	0.005–0.02	0.001	0.003	-0.004–0.009	0.4
**Female sex**	0.2	0.03–0.3	0.02	0.1	-0.01–0.3	0.07
**BMI**	0.01	-0.01–0.02	0.5	0.004	-0.01–0.02	0.6
**Hypertension**	-0.06	-0.3–0.1	0.5	0.1	-0.1–0.3	0.3
**Hyperlipidemia**	0.2	-0.03–0.4	0.1	-0.01	-0.3–0.2	0.9
**Diabetes**	-0.03	-0.2–0.1	0.6	-0.1	-0.2–0.1	0.3
**Smoking**	0.2	0.04–0.3	0.01	0.1	-0.03–0.2	0.1
**Platelet count**	0	-0.001–0.001	0.9	-0.001	-0.002–0.001	0.3
**WBC**	0.04	0.02–0.06	<0.001	0.002	-0.02–0.03	0.8
**Log creatinine**	0.8	0.2–1.4	0.01	0.6	-0.1–1.2	0.07

## Discussion

We found significant associations of IL-6 with *in vivo* P-selectin expression and activation of the fibrinogen receptor GPIIb/IIIa. Moreover, the extent of MPA formation was independently linked to IL-6 suggesting that inflammation increases not only platelet activation but also leukocyte-platelet interaction following angioplasty with stent implantation. ADMA as marker of endothelial dysfunction was significantly associated with activated GPIIb/IIIa. Patients with high IL-6 showed a significantly higher expression of platelet P-selectin, whereas patients with high ADMA exhibited a more pronounced expression of activated GPIIb/IIIa.

Upon platelet activation, P-selectin is released from alpha granules and expressed on the platelet surface. Likewise, the fibrinogen binding site on GPIIb/IIIa becomes exposed [18]. While both P-selectin and activated GPIIb/IIIa are sensitive markers of platelet activation, they represent different properties of activated platelets. Platelet P-selectin is the major ligand for the P-selectin glycoprotein ligand-1 receptor on leukocytes, and mediates the binding of activated platelets to leukocytes [19]. The resulting leukocyte-platelet aggregates can be considered a surrogate marker for platelet activation, and were shown to be elevated in several pathophysiological circumstances, including myocardial infarction [20]. On the other hand, activated GPIIb/IIIa interacts with plasma coagulation and facilitates platelet-platelet interactions.

In our study, we assessed P-selectin expression, activated GPIIb/IIIa and MPA formation without the addition of platelet agonists (= *in vivo*). Since clopidogrel and prasugrel affect mainly adenosine diphosphate (ADP) inducible platelet activation, these parameters should be independent of the type of ADP receptor antagonist. Therefore, we decided to include patients on clopidogrel as well as patients on prasugrel therapy. Indeed, P-selectin expression, activated GPIIb/IIIa and MPA formation did not differ significantly between clopidogrel- and prasugrel-treated patients. Nevertheless, we performed an additional analysis including only clopidogrel-treated patients. However, this did not change the results.

Previous studies reported a worse response to antiplatelet therapy with aspirin and clopidogrel in patients with increased markers of inflammation [5–8]. In detail, IL-6 was found to be an independent predictor of on-treatment residual platelet reactivity in response to arachidonic acid (AA) by light transmission aggregometry (LTA) and of urinary 11-dehydro-thromboxane B2 (D-TXB2) levels [5]. Moreover, hsCRP levels were independent predictors of platelet reactivity when determined by LTA, D-TXB2, the Impact-R and serum thromboxane B2 [5]. Other studies identified IL-6, CRP, WBC and RANTES as independent predictors of on-treatment platelet reactivity to AA and adenosine diphosphate by multiple electrode platelet aggregometry [6–8]. However, all of these studies assessed only agonists’-inducible platelet reactivity. Consequently, data on the association between inflammation and *in vivo* platelet activation were missing, so far. Our findings suggest that the poor response to antiplatelet therapy in patients with increased inflammatory markers may at least in part derive from increased platelet activation *in vivo*.

In a previous publication, supramedian IL-6 levels were independently associated with significantly higher levels of arachidonic acid-inducible platelet reactivity in patients undergoing angioplasty and stenting [5]. Therefore, we decided to use the median as cut-off value for high IL-6 levels.

In our study, only IL-6 was independently associated with both parameters of platelet activation and MPA formation. Other markers of inflammation, i.e. hsCRP and WBC, were not linked to P-selectin expression, activated GPIIb/IIIa and MPA formation. This finding suggests that IL-6 itself may contribute to platelet activation and leukocyte-platelet interaction in atherosclerotic cardiovascular disease. However, it remains to be established whether IL-6 fosters platelet activation, MPA formation and the development of atherosclerotic plaques or is just a surrogate marker for already existing atherosclerosis with ongoing platelet activation.

Upon activation, platelets release more than 300 different bioactive proteins. To the best of our knowledge [21], IL-6 has not been reported to be among these platelet releasates, but there is indirect evidence that platelets have the complete machinery to produce IL-6 [22]. Further, a recent study in mice with dextran sodium sulfate (DSS)-induced colonic inflammation found that the treatment of wild type mice with DSS significantly increased GPIIb/IIIa activation and leukocyte-platelet aggregate formation [23]. In contrast, these platelet responses to DSS were not observed in IL-6 deficient mice. Moreover, chronic IL-6 infusion in wildtype mice reproduced the platelet abnormalities observed in DSS-colitic mice, and IL-6-infused mice also exhibited an acceleration of thrombus formation in their arterioles. In another study, the infusion of IL-6 in normal dogs resulted in an enhanced sensitivity of their platelets to activation with thrombin and platelet-activating factor [24].

It has been reported that *in vitro* IL-6 itself does not induce platelet expression of P-selectin and their aggregation [25]. In contrast, Oleksowicz et al. reported that the incubation of human platelets with IL-6 increased the expression of P-selectin as detected by flow cytometry as well as spheroid and dendritic platelet forms in electron microscopy [26]. Further, they observed an increase in platelet ATP levels after both 1 min and 1 hour IL-6 platelet incubations. Finally, they demonstrated a significant reduction in dense granules in high dose IL-6 incubations by transmission electron microscopy. In a different study, the same group reported that platelet-rich plasma incubated with IL-6 showed a dose-dependent enhancement of agonist-inducible maximal aggregation and secretion of thromboxane B2 [27]. The discrepancy between the different observations may in part be explained by the findings that activated platelets release the soluble IL-6 receptor (sIL-6R), which, in the presence of IL-6 may induce IL-6 trans-signalling, leading to an autocrine activation loop, as evidenced by an increase of gp80 and gp130 content [25]. Recently, high levels of IL-6 were associated with early and late stent thrombosis following percutaneous coronary intervention [28]. Altogether, these findings support the role of IL-6 as mediator or even initiator of platelet activation and MPA formation.

ADMA is an endogenous competitive inhibitor of nitric oxide (NO) synthase. It decreases plasma NO levels and is considered as surrogate marker for endothelial dysfunction. Previously, ADMA was shown to predict cardiovascular and all-cause mortality in patients with angiographic coronary artery disease [29]. In the current study, ADMA was independently associated with the activation of the fibrinogen receptor GPIIb/IIIa and high ADMA levels were linked to a more pronounced expression of activated GPIIb/IIIa. These findings suggest that the interplay of the impaired endothelium with platelets, which are supposed to seal any damage, induces particularly the activation of GPIIb/IIIa, possibly to recruit further platelets from the blood stream.

A higher platelet count was independently associated with a more pronounced formation of MPA. This may be due to the higher number of platelets expressing P-selectin and other cellular adhesion molecules required for the interaction with leukocytes.

A limitation of our study is the lack of clinical outcome data. Moreover, blood sampling was performed one day after the percutaneous procedure, which may affect the extent of platelet activation as well as levels of inflammatory markers.

In conclusion, IL-6 and ADMA are independently associated with platelet activation after percutaneous angioplasty with stent implantation. It remains to be established whether they act prothrombotic and atherogenic themselves or are just surrogate markers for atherosclerosis with concomitant platelet activation.
